# Supplementary material for: Local ancestry inference identifies robust evidence of selection in Neolithic Europe
Source: bioRxiv. 2026 Apr 28:2026.04.23.720248. Preprint. [Version 1] doi: 10.64898/2026.04.23.720248 (PMC13142469; doi:10.64898/2026.04.23.720248)
Supplement: Supplement 1 [file media-1.docx]

**Supplemental Information**

**Local ancestry inference identifies robust evidence of selection in Neolithic Europe**

**Georgia Mies and Iain Mathieson**

**Supplementary Figures**

**
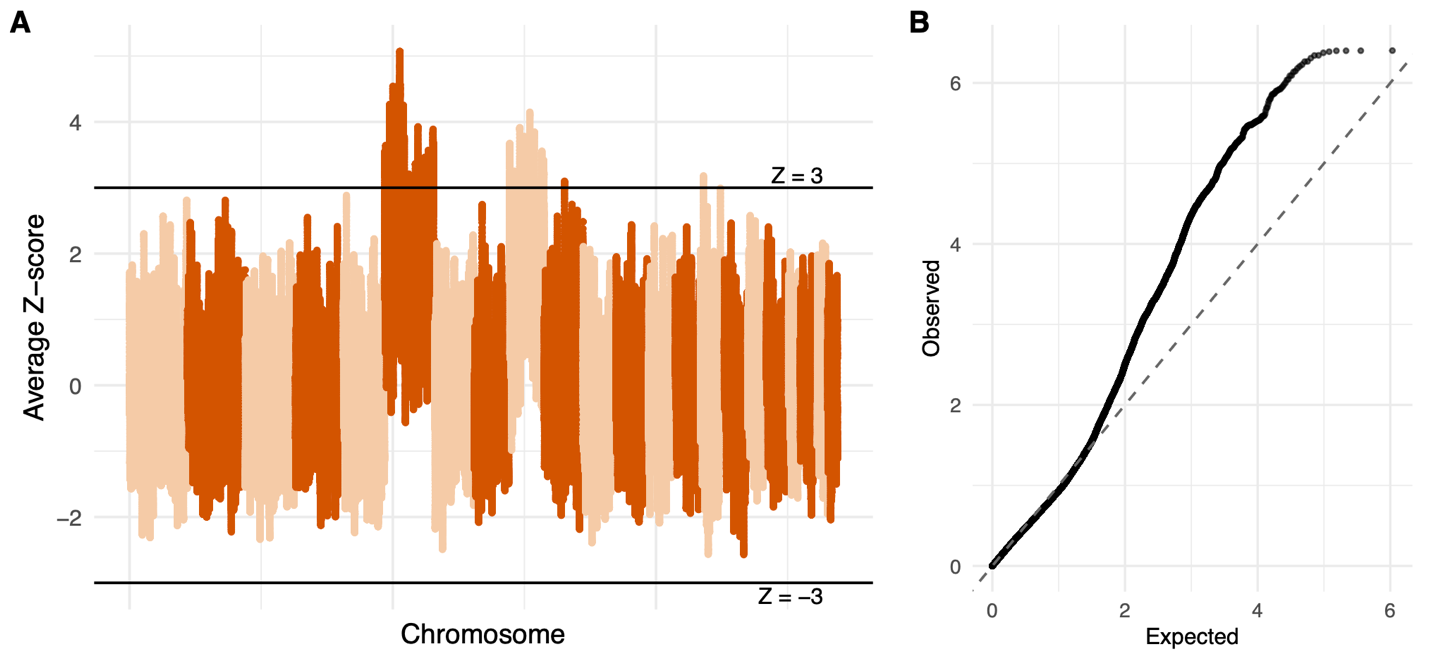
**

**Figure S1. AncestralPaths Z-scores. A)** Manhattan plot showing elevated hunter-gatherer ancestry on chromosomes 6 and 9 prior to per-chromosome normalization to the genome-wide average. **B)** QQ plot of Z-scores.

**
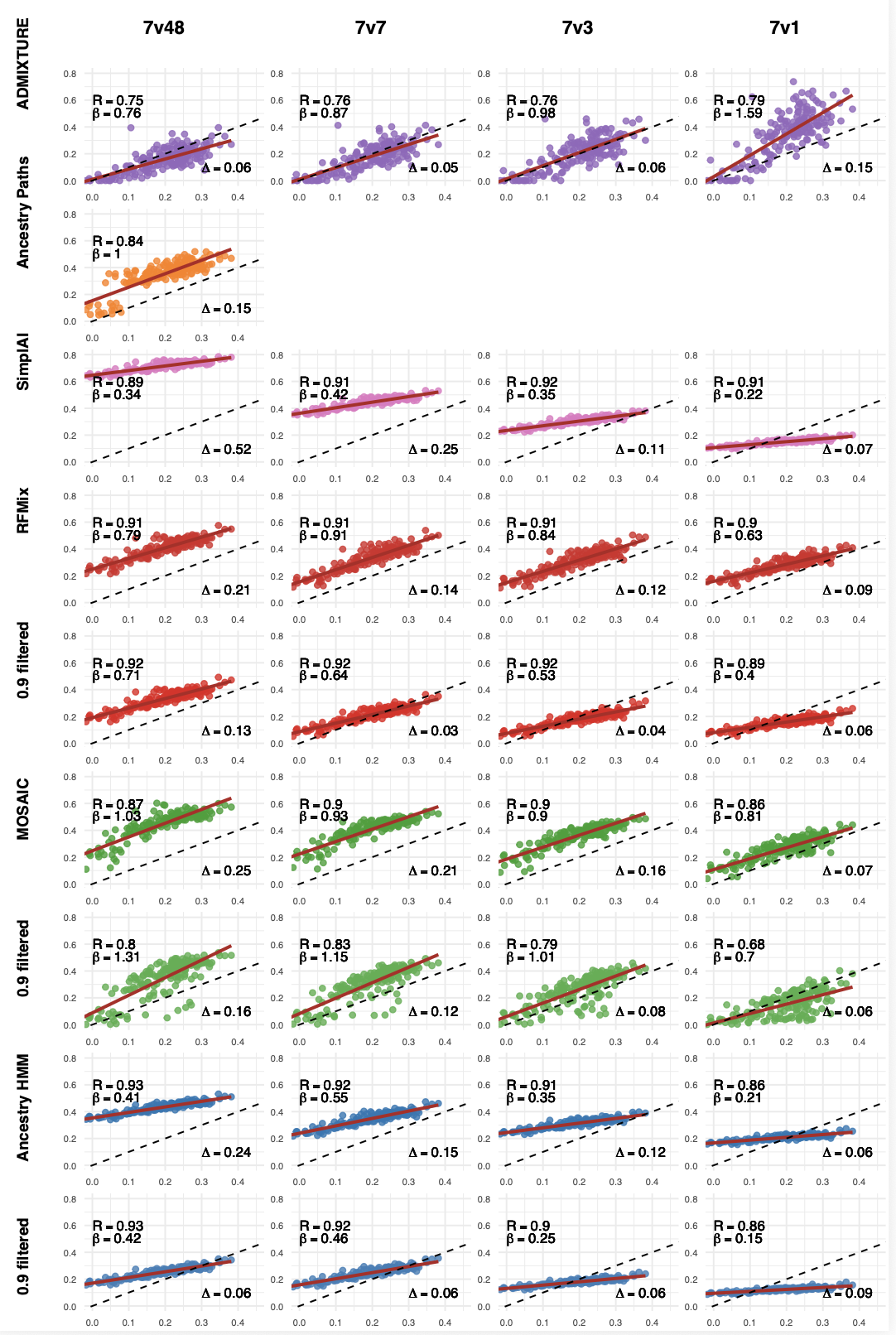
**

**Figure S2. Full genome ancestry estimated by method.** Correlations between global ancestry proportions estimated by ADMIXTURE and LAI methods versus qpAdm. Each point represents one of the 176 admixed Neolithic individuals. R denotes the Pearson correlation between the method and qpAdm individual estimates. β indicates the slope of the regression line between the method and qpAdm estimates. Δ represents the average absolute difference in global ancestry between the LAI method and qpAdm. The dashed line corresponds to y = x for qpAdm estimates, while the red line shows the best-fit linear regression between qpAdm and the LAI method. Sample sizes of sources are listed for each column in the format of 7 farmers v N hunter-gatherers (7vN). Rows are labeled by method and whether calls were filtered by 0.9 posterior probability.

**
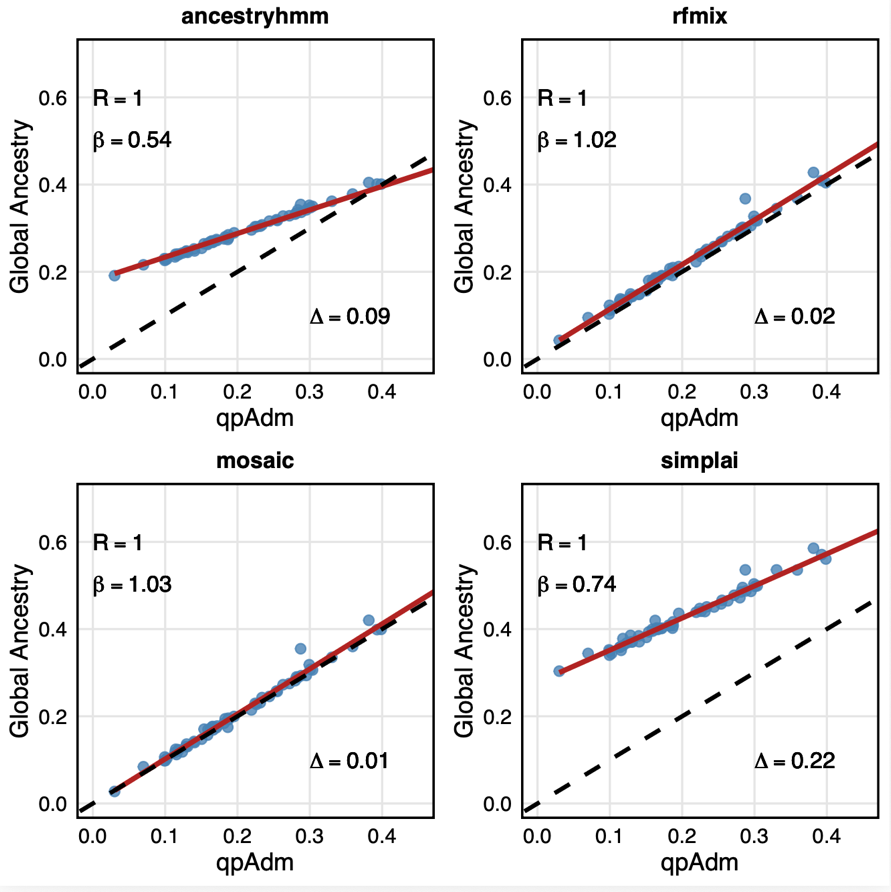
**

**Figure S3. Full genome ancestry estimated by LAI methods in One Thousand Genomes.** Correlations in CEU global ancestry proportions between LAI method and qpAdm. Each point is one of the 61 admixed ASW individuals. R denotes the Pearson correlation between the method and qpAdm individual estimates. β indicates the slope of the regression line between the method and qpAdm estimates. Δ represents the average absolute difference in global ancestry between the LAI method and qpAdm. The dashed line corresponds to y = x for qpAdm estimates, while the red line shows the best-fit linear regression between qpAdm and the LAI method. Sample sizes of sources are 7 CEU and 48 YRI sources for Ancestry HMM, RFMix, and Mosaic, and 7v7 for simpLAI.


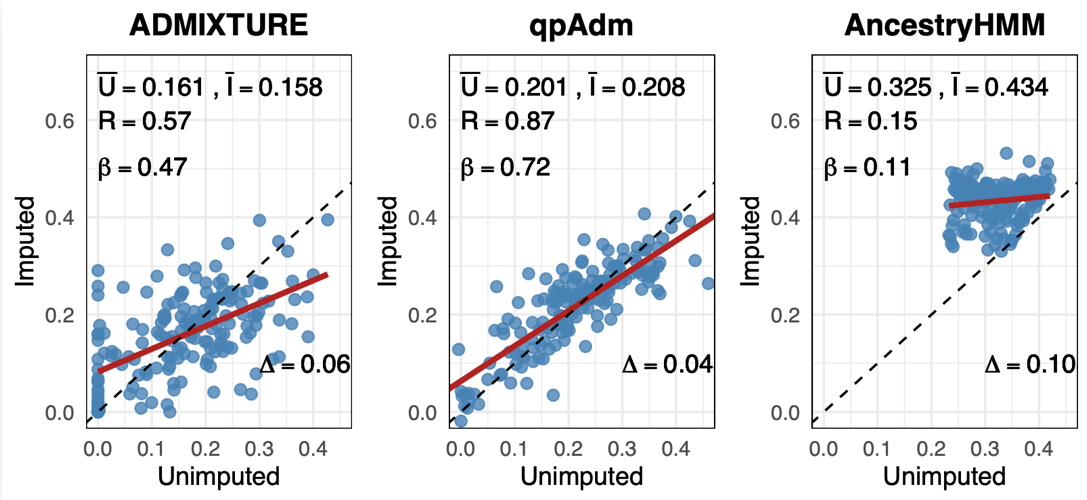


**Figure S4. Full genome ancestry estimated for imputed and unimputed data**. Figures show correlations in hunter-gatherer global ancestry proportions comparing results from **A)** qpAdm, **B)** ADMIXTURE, and **C)** Ancestry HMM on imputed and unimputed data. Each point is one of the 176 admixed Neolithic individuals. Ū and Ī estimates are mean ancestry estimates for unimputed and imputed data for each method respectively. R denotes the Pearson correlation between the method’s individual estimates for unimputed and imputed datatypes. β indicates the slope of the regression line between the method’s two datatypes individual estimates. Δ represents the average absolute difference in global ancestry between the method on each datatype. The dashed line corresponds to y = x for unimputed datatype results, while the red line shows the best-fit linear regression between method on unimputed and imputed data. Sample sizes of sources are 7 farmers and 48 hunter-gatherers.


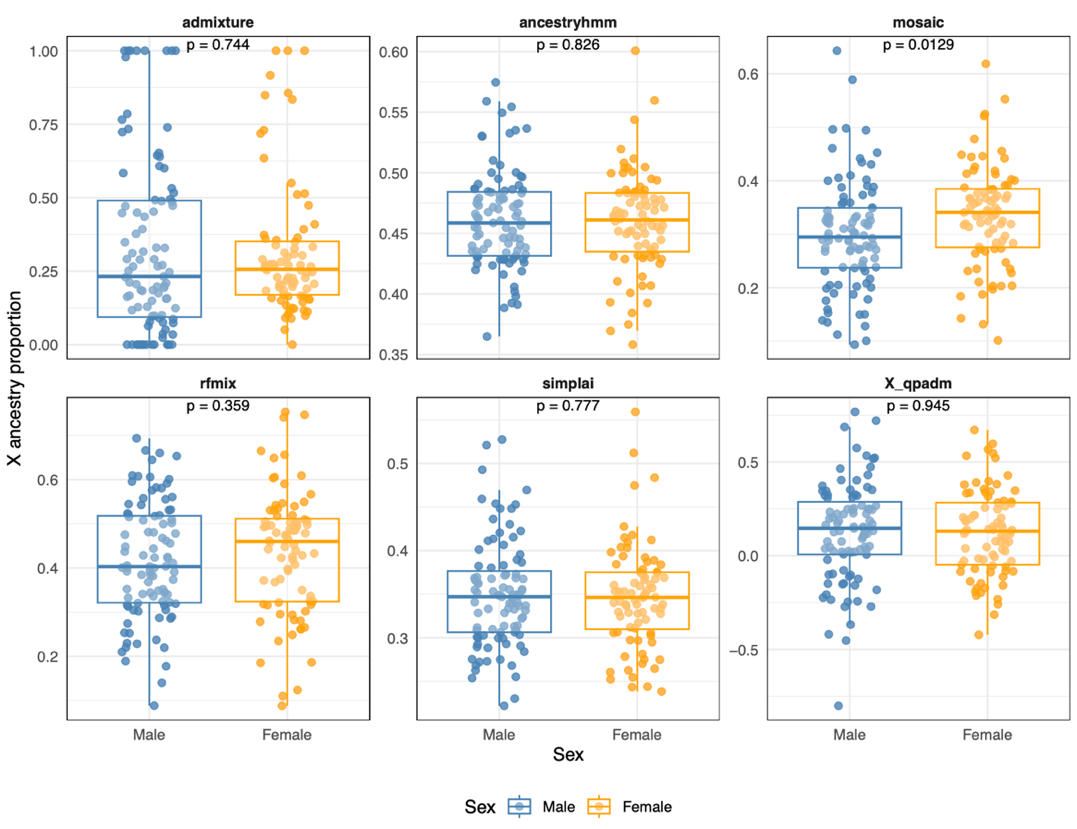


**Figure S5. X chromosome hunter-gatherer ancestry by sex.** Genome-wide X chromosome ancestry proportions for males and females across LAI methods. P-values are from two-sided t-tests comparing mean ancestry between sexes. Boxplots show median and interquartile range.


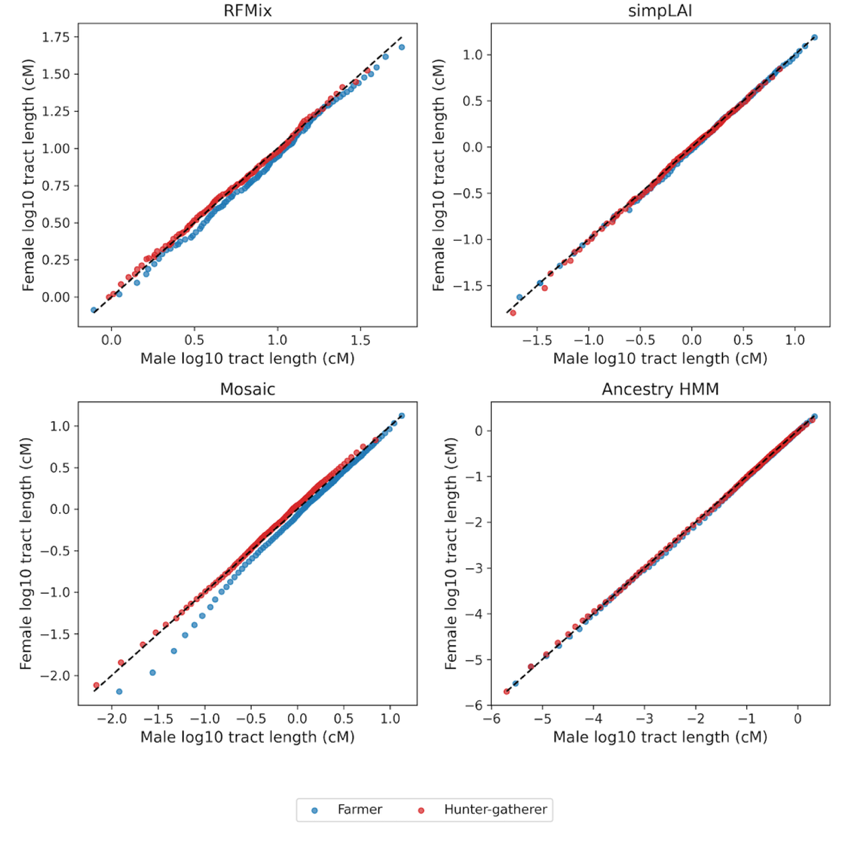


**Figure S6. QQ plot of X chromosome tract length distribution.** Comparing male and female tract length distributions (in log10 tract length (cM)) for each method, shown separately for farmer and hunter-gatherer ancestries.

**
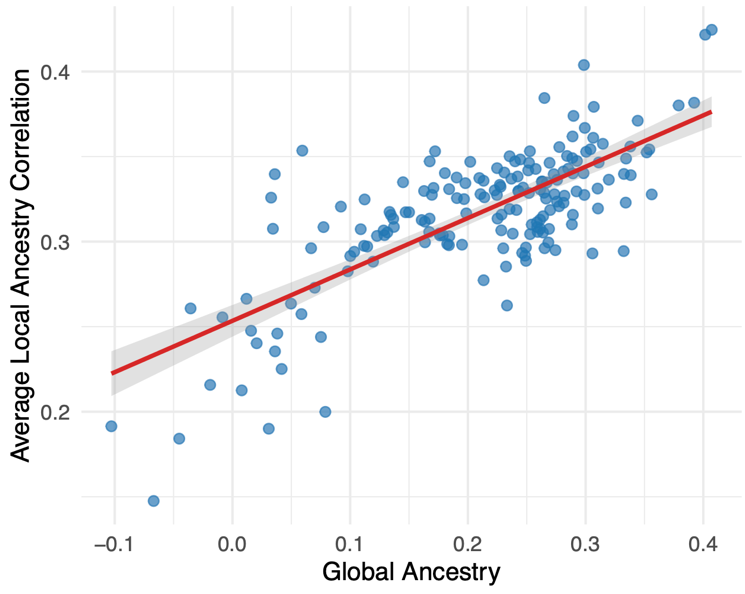
**

**Figure S7. Individual-level average local ancestry correlation across method pairs.** Individual-level average pairwise local ancestry correlation across five methods, computed in 100 kb bins and plotted against global hunter-gatherer ancestry (qpAdm). Each point represents an individual. The red line indicates the best-fit linear regression.

**
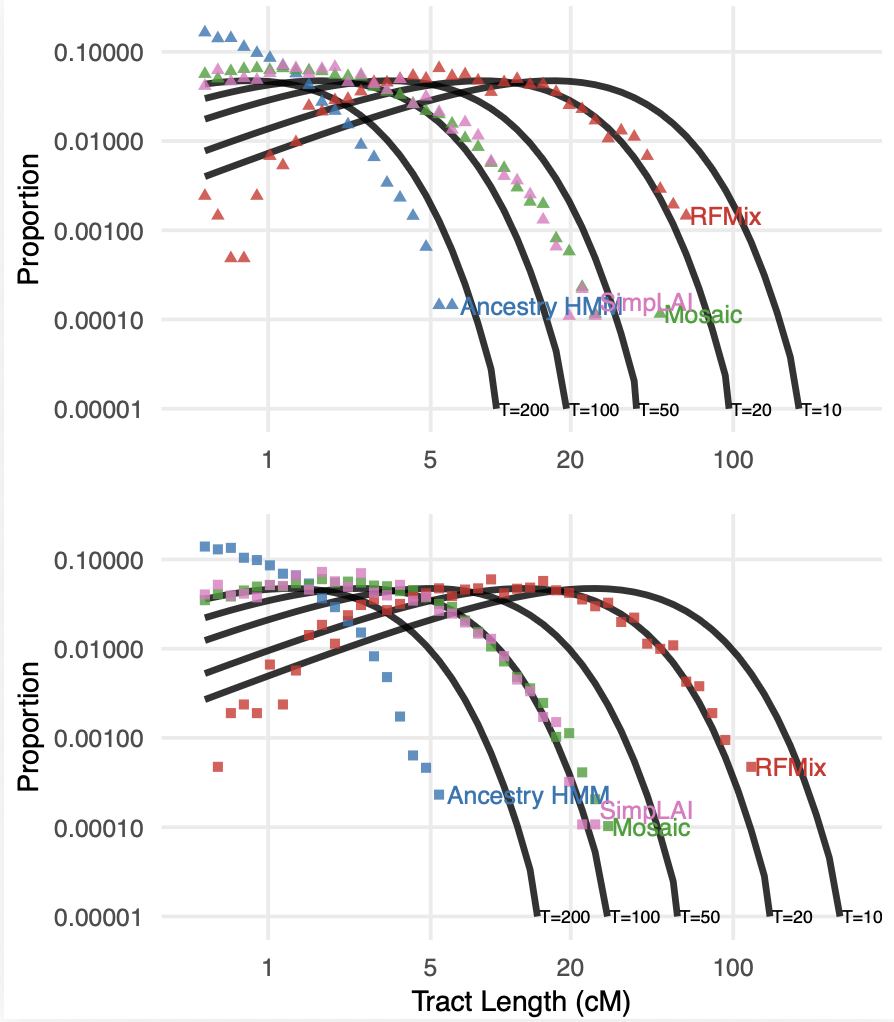
**

**Figure S8. X-chromosome tract length distributions inferred by LAI methods.** Distribution of tract lengths (cM) for each method compared to theoretical expectations (T = 200, 100, 50, 20, 10 generations). Top panel shows hunter-gatherer ancestry tract lengths (triangles) and bottom panel shows farmer ancestry tract lengths (squares).


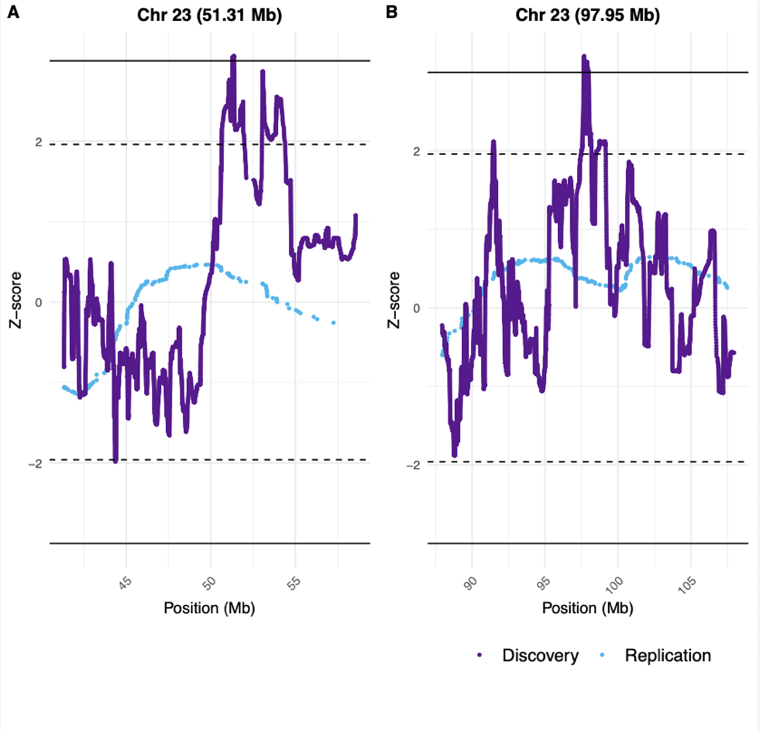


**Figure S9. Replication of X chromosome Z-score signals.** Comparison of combined and replication Z-scores for two X chromosome loci exceeding the Z = ±3 discovery threshold. Solid lines indicate the discovery threshold (Z = ±3), and dashed lines indicate the replication threshold (Z = ±1.96). Discovery results (dark purple) are from RFMix-based X chromosome discovery analyses, and replication results (light blue) are from unimputed replication dataset 1 analyzed with Ancestry HMM.


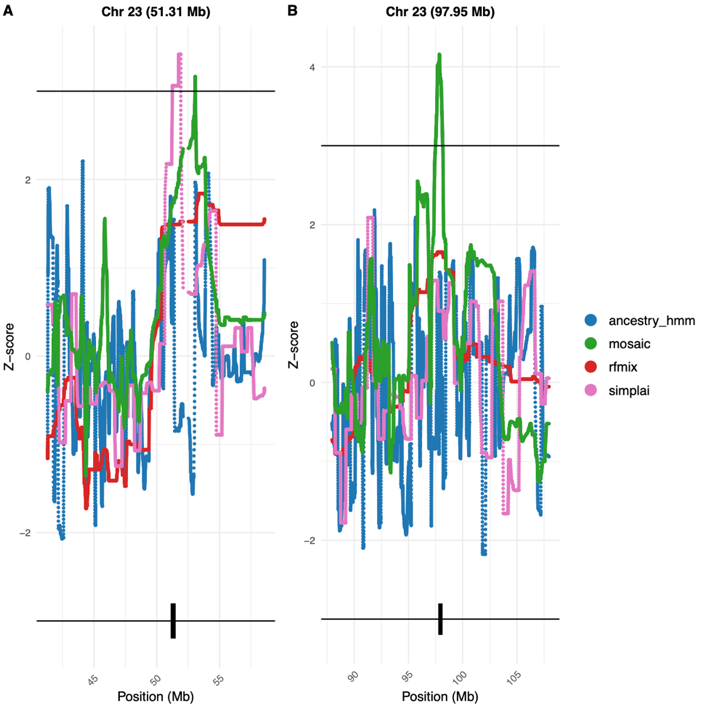


**Figure S10. Z-score deviations across methods for X chromosome loci.** Z-scores across methods for the two discovery X chromosome regions. Horizontal lines indicate Z = ±3 thresholds used in the meta-analysis.


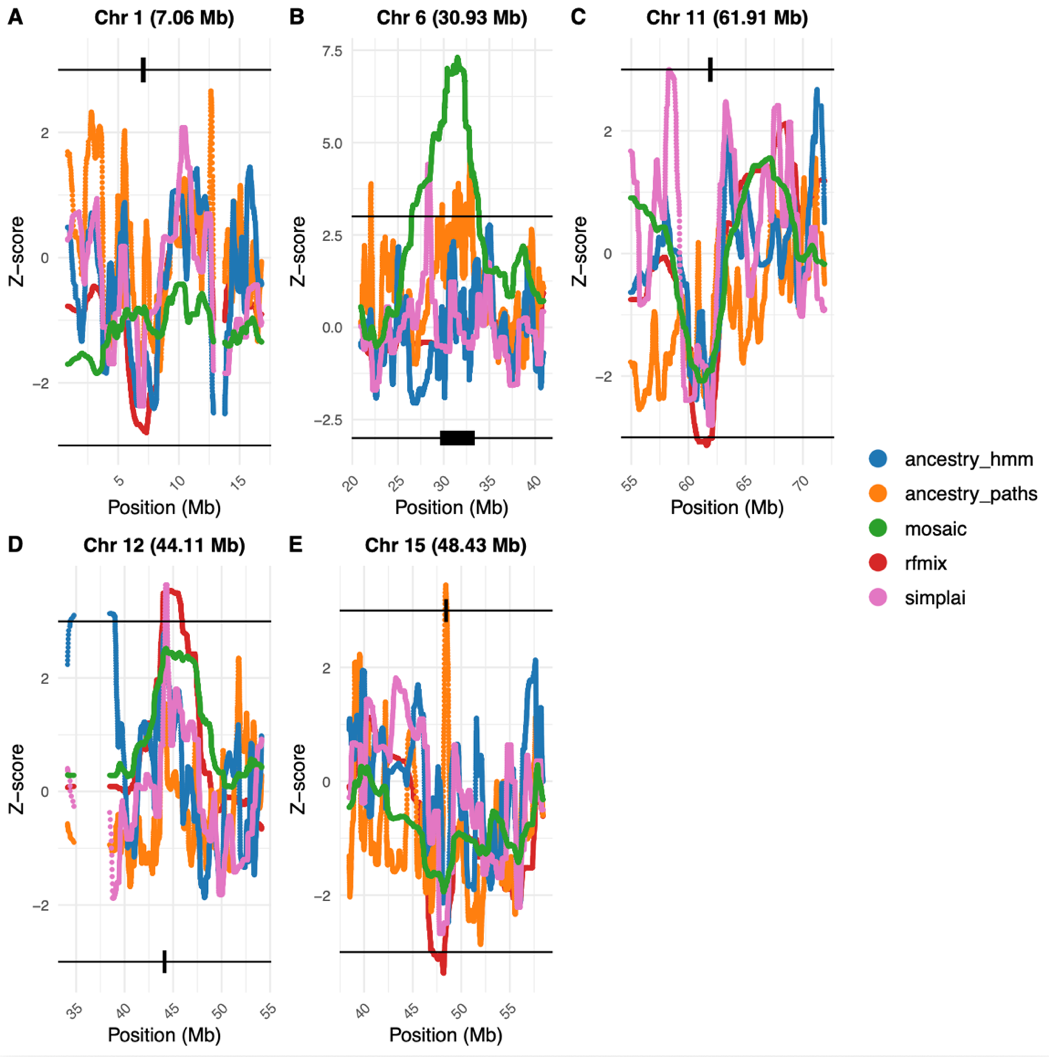


**Figure S11. Z-score deviations across methods for replicated loci.** Z-scores across methods for the five replicated regions. Horizontal lines indicate Z = ±3 thresholds used in the meta-analysis. Methods use 7v48 source panels, except simpLAI (7v7). Putative candidate genes are highlighted with black boxes.


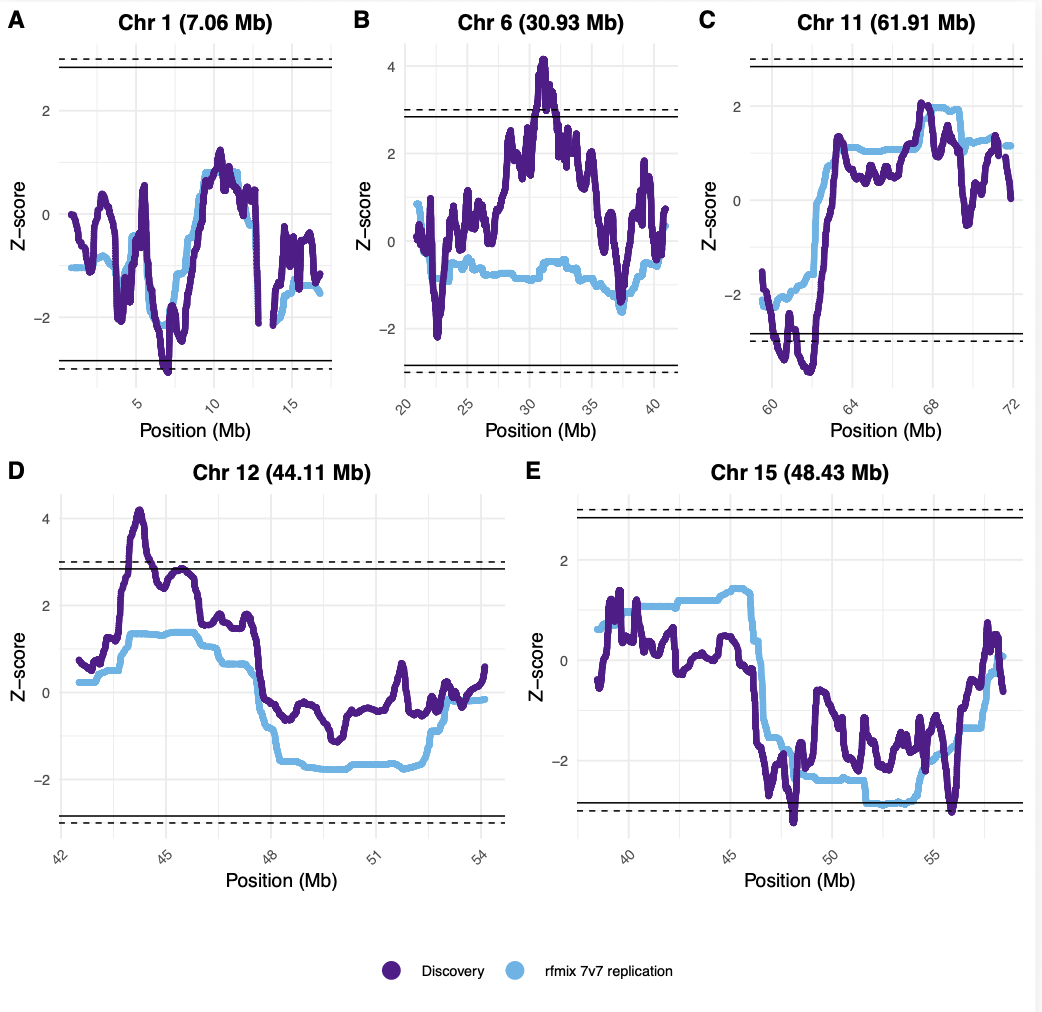


**Figure S12. Replication of Z-scores using RFMix 7v7.** Comparison of combined Z-scores and RFMix 7v7 Z-scores at the five replicated loci. Dashed lines indicate the discovery threshold (Z = ±3), and solid lines indicate the replication threshold (Z = ±2.84).


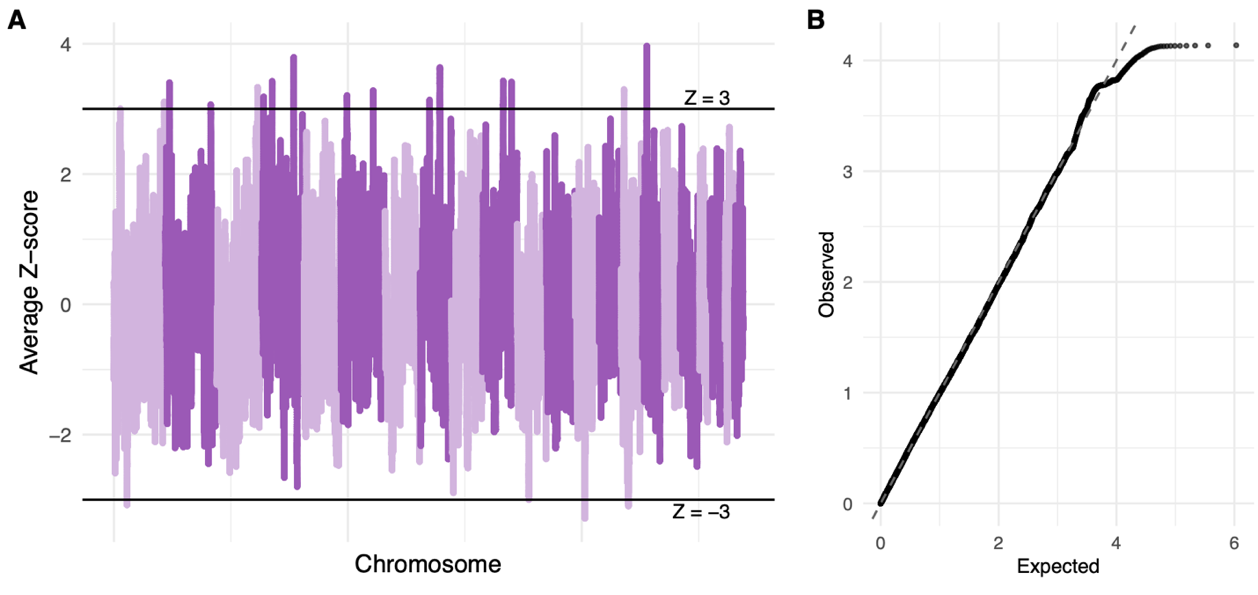


**Figure S13. Manhattan and QQ plots across methods (7vN panels).** Results for all methods using 7vN source panels. Horizontal dashed lines indicate Z = ±3.


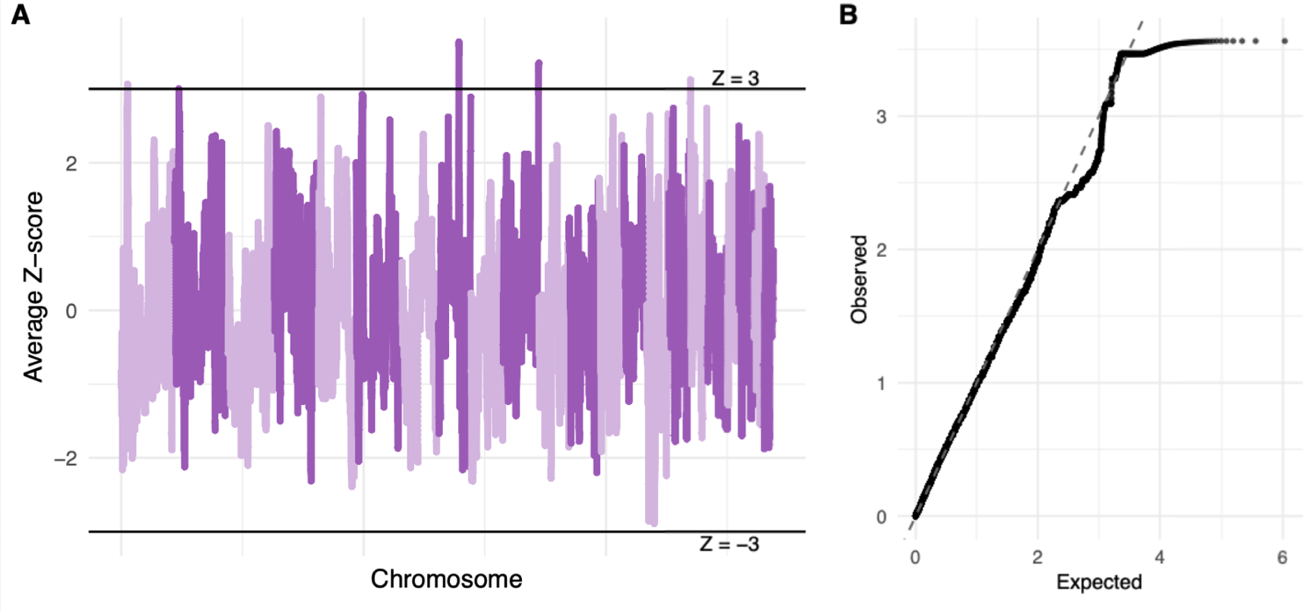
**Figure S14. Manhattan and QQ plots for RFMix 7v7.** Horizontal dashed lines indicate Z = ±3.
